# A UV cross-linking method combined with infrared imaging to analyse RNA–protein interactions

**DOI:** 10.1093/biomethods/bpx009

**Published:** 2017-06-05

**Authors:** Tony Malmqvist, Carl Spickett, Jean-Marc Gallo, Karen Anthony

**Affiliations:** 1 Department of Basic and Clinical Neuroscience, Maurice Wohl Clinical Neuroscience Institute, Institute of Psychiatry, Psychology and Neuroscience, King’s College London, 125 Coldharbour Lane, London SE5 9NU, UK; 2 Atlas Antibodies, Voltavägen 13 A, 16869 Bromma, Sweden; 3 Department of Medical Genetics, University of Cambridge, Cambridge Biomedical Campus, Cambridge CB2 0QQ, UK,; 4 Faculty of Health and Society, University of Northampton, Northampton NN2 7AL, UK

**Keywords:** RNA-binding protein, UV cross-linking, Odyssey®, Tau, HuD, neurodegeneration

## Abstract

Photo cross-linking of proteins with short RNA oligomers is a classical method to study RNA–protein interactions that are implicated in many aspects of RNA metabolism and function. Most commonly, this involves the use of [γ-^32^P]-labeled RNA probes. Although very sensitive, these procedures are complicated by the safety issues associated with the use of radioisotopes. Here, we describe a modified UV cross-linking method using oligonucleotide probes end labelled with the infrared dye IRDye®800. After UV cross-linking, proteins are separated by SDS-PAGE and cross-linked products are visualized with the Odyssey^®^ Infrared Imaging system. This end labelling approach provides a streamlined alternative to random labelling which reduces the efficiency of *in-vitro* transcription. End labelling is also independent of the length of the probe, thus facilitating quantitative comparisons. To validate the method, we have confirmed the binding of HuD to the 3′-UTR of the mRNA for the microtubule-associated protein tau, implicated in the pathogenesis of Alzheimer’s disease. UV cross-linking of HuD with a labeled 21-mer probe was successfully performed using a recombinant purified glutathione-*S*-transferase–HuD fusion protein as well as with lysates from CHO cells transfected with HuD cDNA. UV cross-linking combined with infrared imaging offers a convenient and robust strategy to analyse RNA–protein interactions and their emerging importance in disease.

## Introduction

All aspects of RNA metabolism and function, including pre-mRNA splicing, mRNA stability and subcellular localization are regulated by RNA-binding proteins (RBPs, also referred to as *trans*-acting factors) binding to specific elements in the target RNA [1]. RBPs interact with RNA through specific RNA-binding domains, such as RNA-recognition motifs (RRMs), K-homology domains or zinc fingers [2]. Importantly, abnormal binding of RBPs to RNA has been linked to many pathological conditions, especially neurodegenerative diseases (for reviews see [3, 4]).

Understanding the physiological function and pathological role of RBPs requires a reliable assay to demonstrate binding of specific proteins to RNA substrates. A popular method to study the interaction between an RBP and a specific RNA sequence involves UV cross-linking of the protein with a [γ-^32^P]-labelled RNA probe [5]. UV irradiation induces the formation of a covalent bond between pyrimidine bases in a short (20–25 mer) RNA probe and specific amino acids (e.g. cysteine, lysine, phenylalanine, tryptophan, tyrosine) of an RBP at sites of direct contact with the probe. The covalent RNA–protein bond is maintained after separation by SDS-PAGE allowing detection of labelled RBPs. Although UV cross-linking using radiolabelled probes is very sensitive, the procedure is complicated by the safety issues associated with the use of radioisotopes. An alternative method using near-infrared dye labelled probes has been described, but this method uses a random labelling procedure that reduces the efficiency of *in-vitro* transcription 5-fold in the presence of labelled UTPs [6]. To circumvent this problem, we aimed to validate a streamlined method whereby the RNA probe is commercially end labelled with the infrared dye IRDye^®^800. Cross-linked products are visualized after separation by SDS-PAGE with the Odyssey^®^ Infrared Imaging system from LI-COR Biosciences (Cambridge, UK).

To validate the method, we confirmed the binding of HuD to the 3′-UTR of tau mRNA. Tau is a microtubule-associated protein predominantly expressed in neurons and enriched in axons. Its primary function is to promote microtubule polymerization and stabilization, and it regulates a number of microtubule-dependent processes such as axonal transport [7]. Importantly, tau accumulates in characteristic intracellular filamentous inclusions in several neurodegenerative diseases collectively referred to as the tauopathies (which include Alzheimer’s disease) [8, 9]. Tau mRNA has a long 3′-UTR that contains specific elements regulating mRNA localization and stability. Several *trans*-acting factors that associate with the 3′-UTR of tau mRNA have been identified, including HuD, an embryonic lethal abnormal vision-like family protein with three RRMs, which regulates the stability of tau mRNA [10–12].

UV cross-linking combined with infrared imaging using the Odyssey^®^ system offers a convenient and robust strategy of general applicability to not only demonstrate but also characterize RNA–protein interactions. This procedure will be particularly useful to analyse the properties of RBPs involved in disease, or conversely to confirm the binding of specific RBPs to RNA sequences linked to disease (e.g. [13]).

## Materials and methods

### Expression vectors

cDNA clones for human HuD and rat Tra2β were the generous gifts of Dr Bernard Jasmin (University of Ottawa, Ottawa, Ontario, Canada) and Dr Stefan Stamm (University of Kentucky, Lexington), respectively. cDNAs were subcloned into the expression vector pCMV-tag3B to tag both HuD and Tra2β with a c-myc epitope at their N-terminus. For production of glutathione-*S*-transferase (GST)–HuD fusion protein HuD cDNA was cloned into pGEX-4T-1.

### Production of recombinant GST–HuD fusion protein

The *Escherichiacoli* strain BL21 (DE3) was transformed with pGEX-4T-1 encoding either GST alone or GST–HuD fusion protein. Bacteria were grown at 37°C until an OD_550_ of 0.6–0.9 was reached. Protein expression was induced by adding 1 mM isopropyl β-d-1-thiogalactopyranoside. After overnight incubation at 22°C, bacteria were lysed in lysis buffer [25 mM Tris–HCl, pH 7.4, 0.1 M NaCl, 1 mM ethylenediaminetetraacetic acid (EDTA), lysozyme (0.4 mg/ml), 2 mM phenylmethylsulfonyl fluoride, 1 mM benzamidine, 1 mM Dithiothreitol (DTT) and Complete^®^ mini protease inhibitor cocktail], followed by sonication. After centrifugation, GST–HuD fusion protein was bound to glutathione-4B beads (GE Healthcare Life Sciences, Buckinghamshire, UK), in TNE buffer (25 mM Tris–HCl, pH 7.4, 0.1 M NaCl, 1 mM EDTA and Complete^®^ mini protease inhibitor cocktail), and eluted with 1 ml of elution buffer (20 mM reduced glutathione, 0.2% (v/v) β-mercaptoethanol and 50% (v/v) glycerol in TNE buffer). Protein concentrations were determined using the Bradford protein assay (Bio-Rad Laboratories).

### Cell culture and transfection

CHO cells were cultured in Ham’s F-12 medium supplemented with 10% (v/v) fetal bovine serum, 2 mM l-glutamine and 100 units/ml penicillin. Cells were maintained at 37°C in a humidified atmosphere of 5% CO_2_/95% air. Cells were seeded in 6-well plates and transfected using the jetPEI^TM^ reagent (PolyPlus-transfection, Illkirch, France) according to the manufacturer’s instructions. Cells were analysed 24 h after transfection. After transfection cells were rinsed with phosphate- buffered saline (PBS) at 4 °C and lysed for 30 min in extraction buffer [10 mM HEPES, 3 mM MgCl_2_, 14 mM KCl, 5% (v/v) glycerol, 0.2% (v/v) Nonidet P-40, 1 mM DTT and Complete^®^ Mini protease inhibitor cocktail (Roche)]. The lysates were centrifuged at 10 000 rpm for 5 min at 4°C and the supernatant was collected and protein concentration was measured using the Bradford protein assay (Bio-Rad Laboratories).

### UV cross-linking

The F21 RNA probe (5′-CUUUUUUUUUUUUUACUUUAG-3′) was synthesized with an IRDye^®^ 800CW NHS Ester 5′ modification by Integrated DNA Technologies (Coralville, IA, USA). UV cross-linking was performed as follows: all steps were performed at room temperature and protected from light unless otherwise stated. Recombinant protein (500 ng) or cell lysate (20–40 µg) was incubated with labelled F21 probe (40 pmol) in a reaction volume of 20–30 μl in extraction buffer for 30 min at room temperature. Subsequently, 0.3 U of RNase T1 (Roche Diagnostics Ltd) was added to the RNA–protein mixture and samples were incubated for 10 min, followed by incubation with heparin (5 µg/µl, Sigma–Aldrich) for an additional 10 min at room temperature. The mixture was transferred to a 96-well plate and placed on ice 1–2 cm from the UV light source (254 nm) and irradiated at 3 × 10^5^ µJ in a Stratalinker^®^ UV Crosslinker Model 2400 (Stratagene). RNase A (0.1 µg/µl, Roche Diagnostics Ltd) was then added and the mixture was incubated for 15 min at 37°C. Finally, samples were mixed 1:1 with 2× SDS-PAGE sample buffer and heated at 100°C for 5 min. A total of 10–20 µl of the reactions were loaded on 10% (w/v) polyacrylamide gels and proteins were separated by SDS-PAGE along with Precision Plus Protein Standards (All Blue) (Bio-Rad Laboratories). RNA–protein complexes were detected by scanning the gel at 700 nm and 800 nm using the Odyssey^®^ Infrared Imaging system (LI-COR Biosciences).

### SDS-PAGE and western blotting

For western blotting, proteins separated by SDS-PAGE were transferred to nitrocellulose membranes. After blocking in 5% (w/v) skimmed milk/0.1% (v/v) Tween-20 in PBS for 1 h membranes were incubated with mouse monoclonal anti-c-myc antibody (Sigma) overnight at 4°C in blocking solution followed by AlexaFluor 680 goat anti-mouse IgG (Molecular Probes). Immunoreactivity was detected by scanning membranes at 700 nm using the Odyssey^®^ Infrared Imaging system.

## Results and discussion

**Figure 1:**
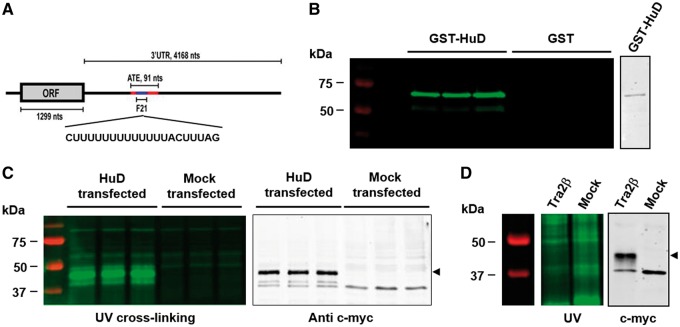
UV cross-linking demonstration of HuD binding to the 3′-UTR of tau mRNA. (**A**) Structure of tau mRNA. The 21 base-long HuD binding site (blue), which is encompassed within the ATE (red) in the 3′-UTR of tau mRNA, was used as an RNA probe (F21). ORF: open reading frame. (**B**) Left panel: UV cross-linking analysis showing a direct binding of the IRdye^®^ 800-labelled F21 probe to recombinant GST–HuD. Right panel: Coomassie blue stained gel of purified GST–HuD fusion protein. (**C**) Analysis of lysates from CHO cells transfected with c-myc-tagged HuD by UV cross-linking with the F21 probe. Binding of the F21 probe to HuD is detected in HuD-transfected cells but not in mock-transfected cells (left panel). Cell lysates were analysed by western blotting with anti-c-myc antibody confirming the molecular weight of HuD (right panel, arrowhead). (**D**) Analysis of lysates from CHO cells transfected with c-myc-tagged Tra2β by UV cross-linking with the F21 probe. Only non-specific background and no binding of the F21 probe to Tra2β were observed (left panel, the gel was intentionally scanned at high resolution to show absence of products co-migrating with Tra2β). Cell lysates were analysed by western blotting with an anti-c-myc antibody confirming expression and molecular weight of Tra2β (right panel, arrowhead). Images are representative of at least three biological replicates.

HuD binds to a U-rich region in the 3′-UTR of tau mRNA, within a 91 base stem loop structure responsible for the selective localization of tau mRNA to the axon. This region is referred to as the axonal-targeting element (ATE) [10, 14–16]. A 21 base-long RNA probe, F21, corresponding to nucleotides 2574–2595 of rat tau mRNA and comprising the U-rich sequence within the ATE in the 3′-UTR of tau mRNA was synthesized and labelled at its 5′-end with IRDye^®^800 fluorochrome (Fig. 1A).

UV cross-linking of HuD with the IRDye^®^800-labelled probe was first performed using a purified recombinant GST–HuD fusion protein. After UV cross-linking, proteins were separated by SDS-PAGE and the gel was scanned directly with the Odyssey^®^ imager. Two fluorescent bands were detected in reactions containing GST–HuD and were absent from reactions containing GST only (Fig. 1B, left panel). The major fluorescent band migrated at the expected molecular weight for GST–HuD (Fig. 1B, right panel) and represents the full-length fusion protein. The minor band likely corresponds to a degradation product of the fusion protein.

To further assess the general applicability of the method, UV cross-linking with the IRDye^®^800-labeled tau probe was performed using lysates from CHO cells transfected with c-myc-tagged HuD cDNA. Mock-transfected cell lysates were used as a negative control. After UV cross-linking, proteins were separated by SDS-PAGE and cross-linked products were visualized as above. A fluorescent band of ∼40 kDa was present in the samples from HuD-transfected cells, but not in samples from mock-transfected cells (Fig. 1C, left panel). This band corresponds to the molecular weight of HuD and co-migrates with HuD detected by western blotting using anti-c-myc antibody (Fig. 1C, right panel).

As a control for the specificity of cross-linking, the same analysis was performed using lysates from CHO cells transfected with another RBP, transformer 2β (Tra2β). Western blotting of CHO cell lysates transfected with c-myc-tagged Tra2β confirmed Tra2β expression, visualized as a single ∼40 kDa band (Fig. 1D, right panel). Fluorescence analysis of the gel did not reveal any interaction between Tra2β and the IRDye^®^800-labelled F21 probe when scanned at high exposure. There was no fluorescent band corresponding to the molecular weight of Tra2β and there were no differences in the background (present as a result of over exposure) observed between Tra2β-transfected cells and mock-transfected cells (Fig. 1D, left panel).

Taken together, we have validated the use of commercially produced end labelled fluorescent probes for UV cross-linking by confirming the specific binding of HuD to the 3′-UTR of tau mRNA. At least three biological replicates were performed during validation. All replicates demonstrated a highly similar and definitive presence/absence of signal to the protein being tested indicating a high reproducibility.

The activity and specificity of RNases in RNA–protein interaction studies is worth careful consideration. We have used RNases with sequence specificity to help avoid over digestion and digestion of the probe. Firstly, before UV irradiation, RNase T1 was used which cleaves specifically at the 3′-end of guanosines (the sequence of our probe contained a single guanosine at the 3′-end). This helps to disrupt large messenger ribonucleoprotein complexes and reduces competition between other abundant RNAs present in the cell lysate. Secondly, after UV irradiation, RNase A was added which cleaves at the 3′-end of pyrimidines to ensure all unbound probe is digested.

The photostability of IRDye^®^ infrared dyes is good-excellent depending on the dye used. Our experience is that there is no significant photobleaching using an Odyssey^®^ infrared scanner and IRDye^®^(s) for UV cross-linking and western blotting applications (where repetitive scanning is not typically required). IRDye^®^ 800CW has a measured fluorescence decay rate of 1.73 kz (bleached dye/s) [17]. Although not currently available for commercial end labelling, the use of extremely photostable IRDye^®^ infrared dyes such as IRDye^®^ 700DX (measured fluorescence decay rate of 0.024 kz bleached dye/s) [17] alongside significantly less photostable dyes may require further consideration for comparative studies. Standard operating procedures should always mitigate against photobleaching by the inclusion of a positive control (e.g. HuD for the 3′-UTR of tau mRNA) and by performing the experiment over a short timeframe in the dark.

As UV cross-linking is typically performed on short RNA probes, end labelling of the probes is the method of choice. End labelling offers several advantages over the random incorporation of labelled UTPs for UV cross-linking applications. Firstly, our streamlined approach removes the difficulties (namely efficiency and reproducibility) associated with *in-vitro* transcribing intact full-length probes in the presence of labelled UTPs [6]. Our method requires minimal optimization and reduces the number of steps and reagent handling. Although a higher degree and specific activity of labelling can be achieved by randomly labelling nucleic acids, with end labelling the intensity of the signal is independent of the length of the probe. This facilitates fully quantitative comparisons and offers a greater applicability for example with hybridization experiments. The wide availability of sensitive fluorescent imagers in effect circumvents the need for the extra intensity that would be afforded by random labelling. In line with others [6], we determined that as little as 80 fmol of IRDye^®^ 800 is required for detection using an Odyssey^®^ infrared scanner. Our method uses 40 pmol of commercially synthesized end-labelled probes, well above the limit of sensitivity. Furthermore, multiple probes would reliably have the same signal strength and sensitivity limit. We demonstrate using the Odyssey^®^ Infrared Imaging system that the IRdye^®^ 800 end labelling of probes is sufficient to identify and characterize RNA–protein interactions. The use of such dyes additionally allows for the identification of multiple RNA–protein interactions from the same sample using alternatively coloured probes.

Our work establishes the value of UV cross-linking with fluorescently end-labelled probes combined with infrared imaging to study RNA–protein interactions. We have streamlined a UV cross-linking method that will be of value to researchers wishing to identify and characterize the emerging role of RNA–protein interactions in disease.

## Author contributions

T.M. performed experiments. C.S. helped in the design of experiments. K.A. and J.-M.G. conceived and planned the study. K.A., T.M. and J.-M.G. wrote the manuscript. The manuscript was reviewed and approved by all authors.

## Funding

This work was supported by the Alzheimer’s Research UK, the Medical Research Council, the Biotechnology and Biological Sciences Research Council and the Psychiatry Research Trust.

*Conflict of interest statement*. Dr Anthony serves on the editorial board of the journal *Biology Methods and Protocols*.
